# Rare gene fusion rearrangement SPTNB1-PDGFRB in an atypical myeloproliferative neoplasm

**DOI:** 10.1186/s13039-018-0405-1

**Published:** 2018-10-19

**Authors:** Vanessa Fiorini Furtado, Neeraj Y. Saini, William Walsh, Venu Bathini, Patricia M. Miron

**Affiliations:** 1 Department of Internal Medicine, University of Massachusetts Medical School, 55 Lake Avenue N, Worcester, MA 01655 USA; 2 Division of Hematology/Oncology, University of Massachusetts Medical School, Worcester, MA USA; 3 Department of Pathology, UmassMemorial Medical Center, Worcester, MA USA

**Keywords:** Myeloproliferative neoplasm, PDGFR mutation

## Abstract

The 2016 revision to the World Health Organization classification of myeloid neoplasms and acute leukemia recognizes a distinct class of myeloid and lymphoid tumors with eosinophilia-related proliferations associated with specific gene rearrangements, one of which involves rearrangements of platelet-derived growth factor receptor B (PDGFRB) gene. We report a case of a rare PDGFRB rearrangement with SPTNB1 (spectrin beta, nonerythrocytic 1) that presented as atypical myeloproliferative neoplasm.

Dear Editor,

The 2016 revision to the World Health Organization classification of myeloid neoplasms and acute leukemia recognizes a distinct class of myeloid and lymphoid tumors with eosinophilia-related proliferations associated with specific gene rearrangements, one of which involves rearrangements of platelet-derived growth factor receptor B (*PDGFRB*) gene [1]. More than 30 fusion partners of *PDGFRB* gene have been reported [2]. Although uncommon, they are important for diagnosis and treatment [3–8]. We report a case of a rare *PDGFRB* rearrangement with *SPTNB1* (spectrin beta, nonerythrocytic 1) that presented as atypical myeloproliferative neoplasm.

A 76-year-old male presented with progressively worsening of dyspnea on exertion and complete blood count revealed macrocytic anemia (hemoglobin 8.3 mg/dl), monocytosis and lymphopenia. Etiology was not delineated at the time, but subsequently the patient became transfusion dependent. His bone marrow was consistent with myeloproliferative disease with hypercellularity and increased myeloid:erythroid ratio of 5:1 with a prominent granulocytic hyperplasia associated with eosinophilia (24%). Remarkably, peripheral blood (PB) eosinophil counts were normal. *BCR-ABL* rearrangement was not detected by fluorescence in situ hybridization (FISH) of PB.

Cytogenetic analysis of bone marrow revealed 16/20 cells to represent an abnormal clone with a (2;5) translocation: 46,XY,t(2;5)(p21;q33)[16]/46,XY [4] (Fig. 1a). Interphase FISH evaluation for *PDGFRB* rearrangement was performed with a PDGFRB Break Apart probe (Kreatech Diagnostics, Inc./Leica Biosystems, Buffalo Grove, IL) at 5q33; rearrangement was observed in 85/100 nuclei (Fig. 1b). Based on a single previous report of a t(2;5)(p21;q33) that was determined to represent an *SPTBN1/PDGFRB* fusion, FISH was performed to assess possible involvement of *SPTBN1.* Two BAC probes, RP11-378O10 and RP11-564H16 (Empire Genomics, Buffalo, NY) that together span a 310 kb region containing *SPTBN1* (Fig. 1d) were hybridized to both metaphase and interphase cells. Interphase FISH showed rearrangement (splitting) of RP11-5644H16 in 75/100 nuclei; metaphase FISH showed RP11-5644H16 to be split with signal on both the derivative 2 and the derivative 5, and RP11-378O10 to be translocated entirely to the derivative chromosome 5 (Fig. 1c). Thus, the chromosome 2 breakpoint is within the *SPTBN1* gene. To our knowledge, this is only the second report of an *SPTBN1/PDGFRB* rearrangement. Of note, rearrangement of *SPTBN1* with other partner genes also has been reported rarely [9, 10].

**Fig. 1:**
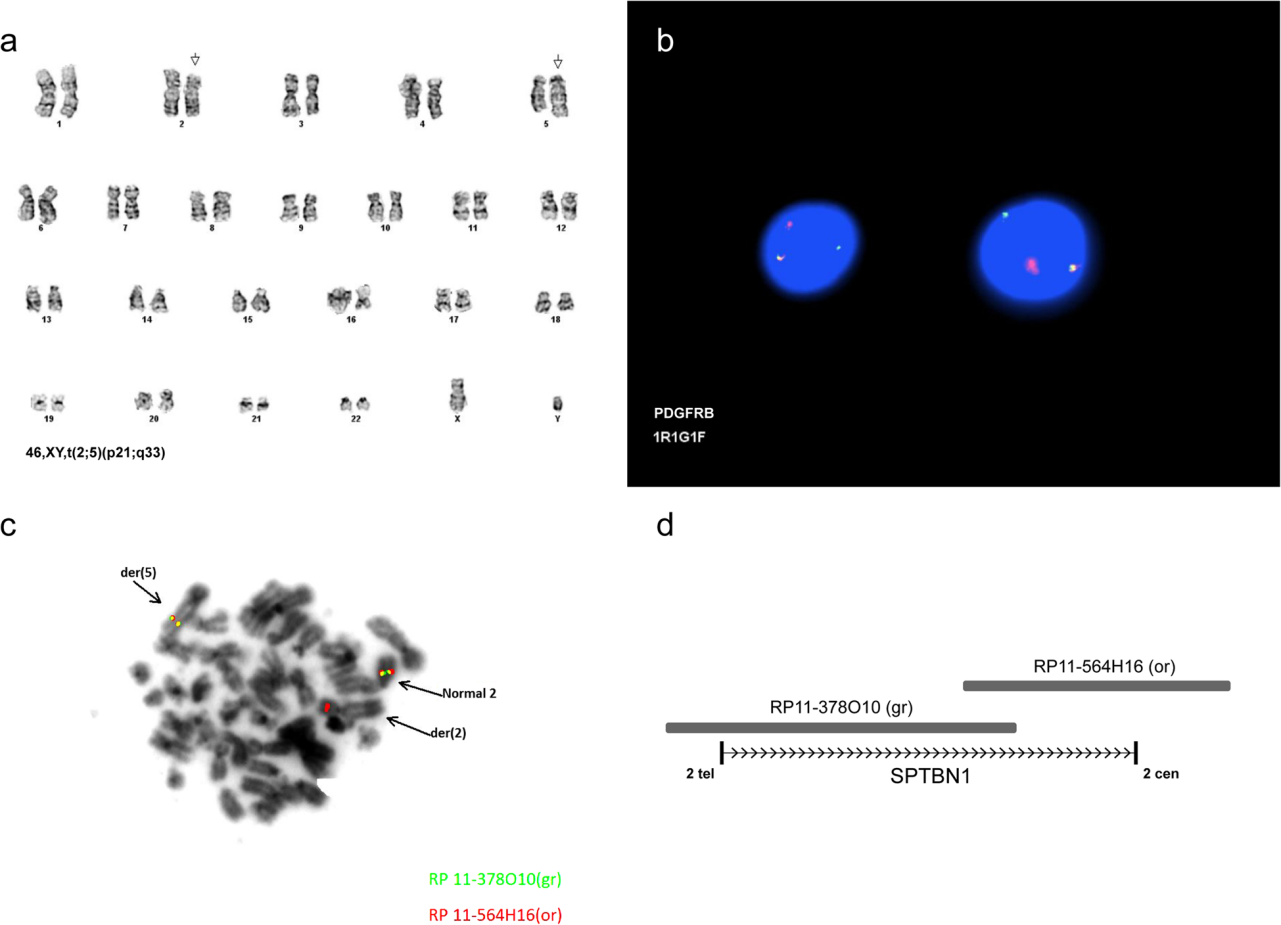
**a** Karyogram showing translocation t(2;5)(p21;q33). **b** FISH analysis with a *PDGFRB* break-apart probe on interphase nuclei showing one normal intact fusion signal and one split signal (one red, one green) confirming *PDGFRB* gene rearrangement. **c** Metaphase FISH showing RP11-5644H16 to be split with signal on both the derivative 2 and the derivative 5, and RP11-378O10 translocated to the derivative chromosome 5. **d** Map of BAC probes relative to *SPTBN1* gene. Direction of *SPTBN1* transcription is towards the centromere on chromosome 2

Imatinib mesylate 200 mg daily was initiated. After 3 months of therapy, patient achieved complete hematological response and became transfusion independent. His dose of imatinib was tapered to 200 mg weekly in 1 year and patient has remained in hematological remission for more than 3 years. Although imatinib was originally designed as a specific inhibitor of the BCR-ABL tyrosine kinase, it has been shown to be effective toward PDGFRB-associated MPN [3, 4, 6, 7]. Prior study reported 10-year OS of 90% in patients with myeloid malignancies bearing *PDGFRB* fusion genes who were treated with imatinib [4]. Furthermore, achievement of rapid and durable complete cytogenetic and molecular responses on doses lower than 400 mg, suggests that patients with *PDGFRB* rearrangements may be more sensitive to imatinib [4]. Our case report highlights the exquisite sensitivity of *PDGFR* gene fusion rearrangement to imatinib in patients with myeloid malignancies and suggests lower weekly doses of imatinib can be considered in this patient group.
